# Supplementary material for: Modeling glioblastoma heterogeneity as a dynamic network of cell states
Source: Mol Syst Biol. 2021 Sep 16;17(9):e10105. doi: 10.15252/msb.202010105 (PMC8444284; doi:10.15252/msb.202010105)
Supplement: Supplementary file 5 — Source Data for Figure 3 [file MSB-17-e10105-s001.zip › Figure3A_sourcedata/GSEA_3065/hallmarks_state5.GseaPreranked.1623416302988/HALLMARK_APICAL_SURFACE.html]

Details for gene set HALLMARK\_APICAL\_SURFACE[GSEA]

|  || Dataset | state5 |
| Phenotype | NoPhenotypeAvailable |
| Upregulated in class | na\_pos |
| GeneSet | HALLMARK\_APICAL\_SURFACE |
| Enrichment Score (ES) | 0.44699252 |
| Normalized Enrichment Score (NES) | 1.2951394 |
| Nominal p-value | 0.122023806 |
| FDR q-value | 0.1531181 |
| FWER p-Value | 0.541 |
Table: GSEA Results Summary

  

Fig 1: Enrichment plot: HALLMARK\_APICAL\_SURFACE      
 Profile of the Running ES Score & Positions of GeneSet Members on the Rank Ordered List

  

| PROBE | GENE SYMBOL | GENE\_TITLE | RANK IN GENE LIST | RANK METRIC SCORE | RUNNING ES | CORE ENRICHMENT || 1 | APP |  |  | 38 | 0.712 | 0.1947 | Yes |
| 2 | BRCA1 |  |  | 522 | 0.305 | 0.2261 | Yes |
| 3 | THY1 |  |  | 546 | 0.299 | 0.3070 | Yes |
| 4 | ADAM10 |  |  | 574 | 0.292 | 0.3856 | Yes |
| 5 | DCBLD2 |  |  | 863 | 0.230 | 0.4178 | Yes |
| 6 | SULF2 |  |  | 1404 | 0.161 | 0.4025 | Yes |
| 7 | SRPX |  |  | 1409 | 0.161 | 0.4470 | Yes |
| 8 | CX3CL1 |  |  | 1776 | 0.128 | 0.4419 | No |
| 9 | B4GALT1 |  |  | 2347 | 0.089 | 0.4032 | No |
| 10 | PLAUR |  |  | 2826 | 0.061 | 0.3670 | No |
| 11 | TMEM8B |  |  | 3294 | 0.037 | 0.3252 | No |
| 12 | ADIPOR2 |  |  | 3460 | 0.029 | 0.3148 | No |
| 13 | GAS1 |  |  | 3841 | 0.014 | 0.2763 | No |
| 14 | NCOA6 |  |  | 4322 | -0.007 | 0.2246 | No |
| 15 | EPHB4 |  |  | 4640 | -0.020 | 0.1948 | No |
| 16 | CRYBG1 |  |  | 5591 | -0.057 | 0.1047 | No |
| 17 | SHROOM2 |  |  | 6849 | -0.125 | -0.0006 | No |
| 18 | GSTM3 |  |  | 7412 | -0.183 | -0.0122 | No |
| 19 | FLOT2 |  |  | 7509 | -0.192 | 0.0307 | No |
| 20 | HSPB1 |  |  | 8586 | -0.478 | 0.0444 | No |
Table: GSEA details [plain text format]

  

Fig 2: HALLMARK\_APICAL\_SURFACE: Random ES distribution      
 Gene set null distribution of ES for **HALLMARK\_APICAL\_SURFACE**
